# [μ-Bis(diphenyl­arsino)methane-1:2κ^2^ *As*:*As*′]nona­carbonyl-1κ^3^ *C*,2κ^3^ *C*,3κ^3^ *C*-[(penta­fluoro­phen­yl)diphenyl­phosphine-3κ*P*]-*triangulo*-triruthenium(0) chloro­form monosolvate

**DOI:** 10.1107/S1600536809055275

**Published:** 2010-01-16

**Authors:** Omar bin Shawkataly, Imthyaz Ahmed Khan, Chin Sing Yeap, Hoong-Kun Fun

**Affiliations:** a Chemical Sciences Programme, School of Distance Education, Universiti Sains Malaysia, 11800 USM, Penang, Malaysia; b X-ray Crystallography Unit, School of Physics, Universiti Sains Malaysia, 11800 USM, Penang, Malaysia

## Abstract

The asymmetric unit of the title *triangulo*-triruthenium compound, [Ru_3_(C_25_H_22_As_2_)(C_18_H_10_F_5_P)(CO)_9_]·CHCl_3_, contains one mol­ecule of the *triangulo*-triruthenium complex and one mol­ecule of the disordered chloro­form solvent. The bis­(diphenyl­arsino)methane ligand bridges an Ru—Ru bond and the monodentate phosphine ligand bonds to the third Ru atom. Both the arsine and phosphine ligands are equatorial with respect to the Ru_3_ triangle. In addition, each Ru atom carries one equatorial and two axial terminal carbonyl ligands. The phosphine-substituted benzene rings make dihedral angles of 68.43 (15), 65.14 (14) and 89.75 (14)° with each other. The dihedral angles between the two benzene rings are 80.70 (15) and 84.53 (16)° for the two diphenyl­arsino groups. In the crystal packing, the mol­ecules are linked into a plane parallel to *bc* by inter­molecular C—H⋯O and C—H⋯F hydrogen bonds. Weak inter­molecular C—H⋯π inter­actions further stabilize the crystal structure.

## Related literature

For general background to *triangulo*-triruthenium derivatives, see: Bruce *et al.* (1985 ▶, 1988*a* ▶,*b* ▶). For related structures, see: Shawkataly *et al.* (1998 ▶, 2004 ▶, 2009 ▶). For the synthesis of μ-bis­(diphenyl­arsino)methane­deca­carbonyl­triruthenium(0), see: Bruce *et al.* (1983 ▶). For the stability of the temperature controller used for the data collection, see: Cosier & Glazer (1986 ▶).
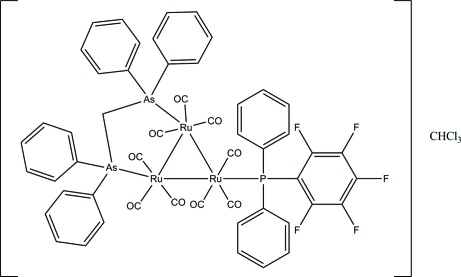


## Experimental

### 

#### Crystal data

[Ru_3_(C_25_H_22_As_2_)(C_18_H_10_F_5_P)(CO)_9_]·CHCl_3_
*M* *_r_* = 1499.16
Triclinic, 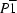

*a* = 12.6354 (3) Å
*b* = 14.1572 (3) Å
*c* = 16.2418 (4) Å
α = 81.497 (1)°
β = 81.452 (1)°
γ = 74.994 (1)°
*V* = 2756.86 (11) Å^3^
*Z* = 2
Mo *K*α radiation
μ = 2.25 mm^−1^
*T* = 100 K
0.46 × 0.20 × 0.14 mm

#### Data collection

Bruker SMART APEXII CCD area-detector diffractometer
Absorption correction: multi-scan (*SADABS*; Bruker, 2005 ▶) *T*_min_ = 0.427, *T*_max_ = 0.745
83481 measured reflections
15949 independent reflections
13916 reflections with *I* > 2σ(*I*)
*R*_int_ = 0.028

#### Refinement

*R*[*F* ^2^ > 2σ(*F* ^2^)] = 0.032
*wR*(*F* ^2^) = 0.088
*S* = 1.07
15949 reflections
702 parameters
6 restraints
H-atom parameters constrained
Δρ_max_ = 2.05 e Å^−3^
Δρ_min_ = −1.70 e Å^−3^

### 

Data collection: *APEX2* (Bruker, 2005 ▶); cell refinement: *SAINT* (Bruker, 2005 ▶); data reduction: *SAINT*; program(s) used to solve structure: *SHELXTL* (Sheldrick, 2008 ▶); program(s) used to refine structure: *SHELXTL*; molecular graphics: *SHELXTL* software used to prepare material for publication: *SHELXTL* and *PLATON* (Spek, 2009 ▶).

## Supplementary Material

Crystal structure: contains datablocks global, I. DOI: 10.1107/S1600536809055275/rz2405sup1.cif

Structure factors: contains datablocks I. DOI: 10.1107/S1600536809055275/rz2405Isup2.hkl

Additional supplementary materials:  crystallographic information; 3D view; checkCIF report

## Figures and Tables

**Table 1:** Hydrogen-bond geometry (Å, °) *Cg*1 and *Cg*2 are the centroids of the C1–C6 and C32–C37 benzene rings, respectively.

*D*—H⋯*A*	*D*—H	H⋯*A*	*D*⋯*A*	*D*—H⋯*A*
C10—H10*A*⋯O5^i^	0.93	2.58	3.469 (4)	160
C12—H12*A*⋯F2^ii^	0.93	2.48	3.274 (3)	143
C22—H22*A*⋯O4^iii^	0.93	2.59	3.451 (5)	154
C34—H34*A*⋯O4^iv^	0.93	2.49	3.201 (4)	134
C39—H39*A*⋯*Cg*1^v^	0.93	2.93	3.749 (3)	148
C41—H41*A*⋯*Cg*2^vi^	0.93	2.73	3.607 (3)	157

Symmetry codes: (i) 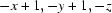; (ii) 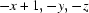; (iii) 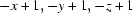; (iv) 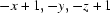; (v) 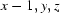; (vi) 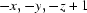.

**Fig. 1.**
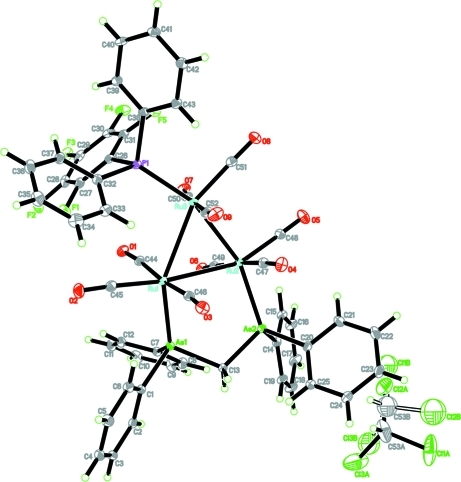
The molecular structure of the title compound with 30% probability ellipsoids for non-H atoms. All disordered components are shown.

**Fig. 2.**
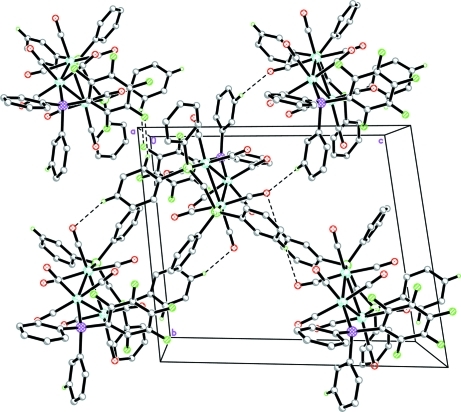
The crystal packing of the title compound, viewed down the a axis, showing the molecules linked into a plane parallel to bc plane. Hydrogen atoms not involved in the hydrogen-bonding (dashed lines) and the solvent molecules have been omitted for clarity.

## supplementary crystallographic information

### Comment

*Triangulo*-triruthenium clusters are known for their interesting structural variations and related catalytic activity. A large number of substituted derivatives, Ru_3_(CO)_12-n_*L*_n_ (*L* = group 15 ligand) have been reported (Bruce *et al.*, 1985, 1988*a*,*b*). As part of our study on the substitution of transition metal-carbonyl clusters with mixed-ligand complexes, we have published several structures of *triangulo*-triruthenium-carbonyl clusters containing mixed P/As and P/Sb ligands (Shawkataly *et al.*, 1998, 2004, 2009). Herein we report the synthesis and structure of title compound.

The asymmetric unit consists of one molecule of the *triangulo*-triruthenium complex and one molecule of disordered chloroform solvent (Fig. 1). The bond lengths and angles of title compound are comparable to those found in its related structure (Shawkataly *et al.*, 2009). The bis(diphenylarsino)methane ligand bridges the Ru1—Ru2 bond and the monodentate phosphine ligand bonds to the Ru3 atom. Both the phosphine and arsine ligands are equatorial with respect to the Ru_3_ triangle. Additionally, each Ru atom carries one equatorial and two axial terminal carbonyl ligands. The phosphine-substituted benzene rings make dihedral angles (C26–C31/C32–C37, C26–C31/C38–C43 and C32–C37/C38–C43) of 68.43 (15), 65.14 (14)and 89.75 (14)° with each other respectively. The dihedral angles between the two benzene rings (C1–C6/C7–C12 and C14–C19/C20–C25) are 80.70 (15) and 84.53 (16)° for the two diphenylarsino groups respectively.

In the crystal packing (Fig. 2), the molecules are linked into plane parallel to *bc* plane by intermolecular C10—H10A···O5, C22—H22A···O4, C34—H34A···O4 and C12—H12A···F2 hydrogen bonds. Weak intermolecular C—H···π interactions further stabilize the crystal structure (Table 1).

### Experimental

All manipulations were performed under a dry, oxygen-free dinitrogen atmosphere using standard Schlenk techniques, all solvents were dried over sodium and distilled from sodium benzophenone ketyl under nitrogen. (Pentafluorophenyl)diphenylphosphine (Maybridge) was used as received and µ-bis(diphenylarsino)methane-decacarbonyl-triruthenium(0) (Bruce *et al.*, 1983) were prepared by a reported procedure. The title compound was obtained by refluxing equimolar quantities of Ru_3_(CO)_10_(µ-Ph_2_AsCH_2_AsPh_2_) (105.5 mg, 0.1 mmol) and (pentafluorophenyl)diphenylphosphine (35.22 mg, 0.1 mmol) in hexane under nitrogen atmosphere. Crystals suitable for X-ray diffraction were grown by slow solvent / solvent diffusion of C_6_H_14_ into CH_2_Cl_2_.

### Refinement

All hydrogen atoms were positioned geometrically and refined using a riding model with C—H = 0.93–0.97 Å and *U*_iso_(H) = 1.2 *U*_eq_(C). The chloroform molecule is disordered over two position with the site occupancies are fixed to 0.50 for both components at final refinement. The disordered components were subjected to rigid bond restraint. The minor component was refined isotropically. The maximum and minimum residual electron density peaks of 2.05 and -1.70 e Å^-3^, respectively, were located 0.44 Å and 0.51 Å from the Cl2B atom.

### Crystal data

**Table:**

[Ru_3_(C_25_H_22_As_2_)(C_18_H_10_F_5_P)(CO)_9_]·CHCl_3_	*Z* = 2
*M**_r_* = 1499.16	*F*(000) = 1464
Triclinic, *P*1	*D*_x_ = 1.806 Mg m^−^^3^
Hall symbol: -P 1	Mo *K*α radiation, λ = 0.71073 Å
*a* = 12.6354 (3) Å	Cell parameters from 9981 reflections
*b* = 14.1572 (3) Å	θ = 2.5–35.0°
*c* = 16.2418 (4) Å	µ = 2.25 mm^−^^1^
α = 81.497 (1)°	*T* = 100 K
β = 81.452 (1)°	Block, red
γ = 74.994 (1)°	0.46 × 0.20 × 0.14 mm
*V* = 2756.86 (11) Å^3^	

### Data collection

**Table:**

Bruker SMART APEXII CCD area-detector diffractometer	15949 independent reflections
Radiation source: fine-focus sealed tube	13916 reflections with *I* > 2σ(*I*)
graphite	*R*_int_ = 0.028
φ and ω scans	θ_max_ = 30.0°, θ_min_ = 1.3°
Absorption correction: multi-scan (*SADABS*; Bruker, 2005)	*h* = −17→17
*T*_min_ = 0.427, *T*_max_ = 0.745	*k* = −19→19
83481 measured reflections	*l* = −22→22

### Refinement

**Table:**

Refinement on *F*^2^	Primary atom site location: structure-invariant direct methods
Least-squares matrix: full	Secondary atom site location: difference Fourier map
*R*[*F*^2^ > 2σ(*F*^2^)] = 0.032	Hydrogen site location: inferred from neighbouring sites
*wR*(*F*^2^) = 0.088	H-atom parameters constrained
*S* = 1.07	*w* = 1/[σ^2^(*F*_o_^2^) + (0.0344*P*)^2^ + 8.2555*P*] where *P* = (*F*_o_^2^ + 2*F*_c_^2^)/3
15949 reflections	(Δ/σ)_max_ = 0.001
702 parameters	Δρ_max_ = 2.05 e Å^−^^3^
6 restraints	Δρ_min_ = −1.69 e Å^−^^3^

### Special details

**Table:**

Experimental. The crystal was placed in the cold stream of an Oxford Cyrosystems Cobra open-flow nitrogen cryostat (Cosier & Glazer, 1986) operating at 100.0 (1) K.
Geometry. All e.s.d.'s (except the e.s.d. in the dihedral angle between two l.s. planes) are estimated using the full covariance matrix. The cell e.s.d.'s are taken into account individually in the estimation of e.s.d.'s in distances, angles and torsion angles; correlations between e.s.d.'s in cell parameters are only used when they are defined by crystal symmetry. An approximate (isotropic) treatment of cell e.s.d.'s is used for estimating e.s.d.'s involving l.s. planes.
Refinement. Refinement of *F*^2^ against ALL reflections. The weighted *R*-factor *wR* and goodness of fit *S* are based on *F*^2^, conventional *R*-factors *R* are based on *F*, with *F* set to zero for negative *F*^2^. The threshold expression of *F*^2^ > σ(*F*^2^) is used only for calculating *R*-factors(gt) *etc*. and is not relevant to the choice of reflections for refinement. *R*-factors based on *F*^2^ are statistically about twice as large as those based on *F*, and *R*- factors based on ALL data will be even larger.

### Fractional atomic coordinates and isotropic or equivalent isotropic displacement parameters (Å^2^)

**Table:**

	*x*	*y*	*z*	*U*_iso_*/*U*_eq_	Occ. (<1)
Ru1	0.524202 (17)	0.149640 (15)	0.235137 (13)	0.01477 (5)	
Ru2	0.464067 (17)	0.348007 (15)	0.276292 (13)	0.01422 (5)	
Ru3	0.306485 (17)	0.231861 (16)	0.312682 (14)	0.01602 (5)	
As1	0.69998 (2)	0.17494 (2)	0.166538 (17)	0.01419 (6)	
As2	0.64874 (2)	0.37420 (2)	0.256950 (17)	0.01438 (6)	
P1	0.21075 (6)	0.11577 (5)	0.29766 (4)	0.01570 (13)	
F1	0.32445 (16)	0.01111 (13)	0.13905 (11)	0.0256 (4)	
F2	0.2792 (2)	0.06177 (15)	−0.01870 (12)	0.0350 (5)	
F3	0.10632 (19)	0.21441 (16)	−0.05752 (12)	0.0338 (5)	
F4	−0.01665 (16)	0.32048 (16)	0.06474 (13)	0.0325 (4)	
F5	0.02756 (14)	0.27309 (14)	0.22319 (11)	0.0247 (4)	
O1	0.41632 (18)	0.19539 (17)	0.07211 (14)	0.0251 (4)	
O2	0.5609 (2)	−0.06487 (17)	0.20836 (17)	0.0336 (5)	
O3	0.61565 (19)	0.08889 (18)	0.40559 (14)	0.0282 (5)	
O4	0.4936 (2)	0.28944 (17)	0.46205 (14)	0.0284 (5)	
O5	0.3194 (2)	0.54971 (17)	0.30900 (15)	0.0319 (5)	
O6	0.45619 (19)	0.40229 (17)	0.08624 (14)	0.0253 (4)	
O7	0.23052 (18)	0.35871 (17)	0.15241 (15)	0.0270 (5)	
O8	0.1254 (2)	0.3807 (2)	0.40125 (19)	0.0401 (6)	
O9	0.3746 (2)	0.1271 (2)	0.48352 (15)	0.0324 (5)	
C1	0.8252 (2)	0.0604 (2)	0.16541 (18)	0.0178 (5)	
C2	0.9180 (2)	0.0583 (2)	0.10684 (18)	0.0196 (5)	
H2A	0.9202	0.1114	0.0660	0.023*	
C3	1.0078 (2)	−0.0238 (2)	0.1095 (2)	0.0233 (6)	
H3A	1.0696	−0.0251	0.0702	0.028*	
C4	1.0053 (3)	−0.1031 (2)	0.1703 (2)	0.0254 (6)	
H4A	1.0650	−0.1577	0.1719	0.030*	
C5	0.9128 (3)	−0.1009 (2)	0.2292 (2)	0.0268 (6)	
H5A	0.9112	−0.1537	0.2704	0.032*	
C6	0.8228 (2)	−0.0199 (2)	0.22656 (19)	0.0216 (5)	
H6A	0.7608	−0.0191	0.2656	0.026*	
C7	0.7080 (2)	0.2292 (2)	0.04967 (16)	0.0168 (5)	
C8	0.7381 (3)	0.3180 (2)	0.02187 (19)	0.0232 (6)	
H8A	0.7572	0.3525	0.0595	0.028*	
C9	0.7392 (3)	0.3543 (2)	−0.0628 (2)	0.0289 (7)	
H9A	0.7587	0.4135	−0.0815	0.035*	
C10	0.7116 (3)	0.3027 (2)	−0.11942 (19)	0.0281 (7)	
H10A	0.7132	0.3270	−0.1759	0.034*	
C11	0.6815 (3)	0.2148 (2)	−0.09154 (19)	0.0259 (6)	
H11A	0.6632	0.1801	−0.1295	0.031*	
C12	0.6786 (2)	0.1783 (2)	−0.00691 (18)	0.0211 (5)	
H12A	0.6572	0.1200	0.0117	0.025*	
C13	0.7620 (2)	0.2601 (2)	0.22097 (17)	0.0162 (5)	
H13A	0.7959	0.2224	0.2693	0.019*	
H13B	0.8190	0.2825	0.1822	0.019*	
C14	0.6792 (2)	0.4828 (2)	0.17732 (17)	0.0175 (5)	
C15	0.5925 (2)	0.5543 (2)	0.14568 (18)	0.0212 (5)	
H15A	0.5202	0.5506	0.1637	0.025*	
C16	0.6136 (3)	0.6314 (2)	0.0871 (2)	0.0256 (6)	
H16A	0.5555	0.6792	0.0659	0.031*	
C17	0.7214 (3)	0.6368 (3)	0.0605 (2)	0.0289 (7)	
H17A	0.7356	0.6877	0.0207	0.035*	
C18	0.8085 (3)	0.5662 (3)	0.0930 (2)	0.0277 (6)	
H18A	0.8808	0.5701	0.0750	0.033*	
C19	0.7876 (2)	0.4900 (2)	0.1523 (2)	0.0222 (6)	
H19A	0.8456	0.4438	0.1753	0.027*	
C20	0.7044 (2)	0.4011 (2)	0.35381 (17)	0.0181 (5)	
C21	0.6342 (3)	0.4680 (3)	0.4041 (2)	0.0320 (7)	
H21A	0.5621	0.4957	0.3921	0.038*	
C22	0.6710 (3)	0.4935 (3)	0.4722 (3)	0.0400 (9)	
H22A	0.6235	0.5380	0.5059	0.048*	
C23	0.7785 (3)	0.4529 (3)	0.4900 (2)	0.0295 (7)	
H23A	0.8032	0.4707	0.5352	0.035*	
C24	0.8481 (3)	0.3866 (2)	0.4410 (2)	0.0257 (6)	
H24A	0.9201	0.3593	0.4532	0.031*	
C25	0.8117 (2)	0.3596 (2)	0.37293 (19)	0.0219 (5)	
H25A	0.8591	0.3139	0.3403	0.026*	
C26	0.1728 (2)	0.1353 (2)	0.19005 (17)	0.0180 (5)	
C27	0.2360 (2)	0.0849 (2)	0.12485 (18)	0.0205 (5)	
C28	0.2146 (3)	0.1108 (2)	0.04191 (18)	0.0244 (6)	
C29	0.1278 (3)	0.1885 (2)	0.02168 (19)	0.0257 (6)	
C30	0.0648 (2)	0.2423 (2)	0.08410 (19)	0.0234 (6)	
C31	0.0880 (2)	0.2150 (2)	0.16600 (18)	0.0199 (5)	
C32	0.2689 (2)	−0.0174 (2)	0.31666 (17)	0.0187 (5)	
C33	0.3620 (3)	−0.0532 (2)	0.35865 (19)	0.0238 (6)	
H33A	0.3986	−0.0095	0.3723	0.029*	
C34	0.4008 (3)	−0.1537 (3)	0.3805 (2)	0.0303 (7)	
H34A	0.4637	−0.1768	0.4079	0.036*	
C35	0.3467 (3)	−0.2193 (2)	0.3617 (2)	0.0305 (7)	
H35A	0.3723	−0.2865	0.3771	0.037*	
C36	0.2530 (3)	−0.1846 (2)	0.3193 (2)	0.0269 (6)	
H36A	0.2164	−0.2287	0.3064	0.032*	
C37	0.2147 (3)	−0.0846 (2)	0.29670 (18)	0.0223 (6)	
H37A	0.1527	−0.0619	0.2681	0.027*	
C38	0.0781 (2)	0.1203 (2)	0.36324 (17)	0.0179 (5)	
C39	−0.0093 (2)	0.0919 (2)	0.33857 (18)	0.0196 (5)	
H39A	−0.0049	0.0762	0.2844	0.023*	
C40	−0.1028 (2)	0.0870 (2)	0.39465 (19)	0.0213 (5)	
H40A	−0.1604	0.0676	0.3778	0.026*	
C41	−0.1109 (2)	0.1109 (2)	0.4755 (2)	0.0241 (6)	
H41A	−0.1740	0.1084	0.5124	0.029*	
C42	−0.0244 (3)	0.1386 (2)	0.50100 (18)	0.0233 (6)	
H42A	−0.0290	0.1537	0.5554	0.028*	
C43	0.0695 (2)	0.1438 (2)	0.44474 (18)	0.0206 (5)	
H43A	0.1269	0.1631	0.4618	0.025*	
C44	0.4530 (2)	0.1837 (2)	0.13446 (18)	0.0194 (5)	
C45	0.5443 (2)	0.0156 (2)	0.21989 (19)	0.0225 (6)	
C46	0.5774 (2)	0.1157 (2)	0.34376 (18)	0.0201 (5)	
C47	0.4802 (2)	0.3059 (2)	0.39310 (19)	0.0205 (5)	
C48	0.3746 (2)	0.4735 (2)	0.29795 (17)	0.0201 (5)	
C49	0.4575 (2)	0.3773 (2)	0.15634 (18)	0.0190 (5)	
C50	0.2671 (2)	0.3107 (2)	0.20913 (19)	0.0216 (5)	
C51	0.1929 (3)	0.3231 (2)	0.3690 (2)	0.0253 (6)	
C52	0.3560 (2)	0.1641 (2)	0.41851 (19)	0.0225 (6)	
Cl1A	0.9948 (5)	0.6545 (2)	0.3796 (2)	0.1120 (18)	0.50
Cl2A	0.8101 (3)	0.6591 (3)	0.2961 (3)	0.0877 (11)	0.50
Cl3A	1.0299 (3)	0.5688 (6)	0.2281 (2)	0.128 (2)	0.50
C53A	0.9402 (11)	0.5940 (8)	0.3180 (6)	0.071 (4)	0.50
H53A	0.9334	0.5310	0.3497	0.086*	0.50
Cl1B	0.7461 (4)	0.7106 (3)	0.2760 (3)	0.0970 (12)*	0.50
Cl2B	0.8857 (6)	0.6721 (5)	0.4055 (4)	0.140 (2)*	0.50
Cl3B	0.9765 (4)	0.6119 (4)	0.2435 (3)	0.1025 (13)*	0.50
C53B	0.8682 (13)	0.6310 (13)	0.3144 (10)	0.089 (5)*	0.50
H53B	0.8512	0.5667	0.3316	0.107*	0.50

### Atomic displacement parameters (Å^2^)

**Table:**

	*U*^11^	*U*^22^	*U*^33^	*U*^12^	*U*^13^	*U*^23^
Ru1	0.01463 (9)	0.01510 (9)	0.01545 (10)	−0.00625 (7)	−0.00122 (7)	−0.00026 (7)
Ru2	0.01310 (9)	0.01471 (9)	0.01522 (9)	−0.00497 (7)	−0.00170 (7)	0.00006 (7)
Ru3	0.01377 (9)	0.01956 (10)	0.01610 (10)	−0.00761 (8)	−0.00084 (7)	−0.00084 (8)
As1	0.01427 (12)	0.01494 (12)	0.01388 (12)	−0.00502 (9)	−0.00136 (9)	−0.00087 (9)
As2	0.01357 (12)	0.01500 (12)	0.01544 (12)	−0.00520 (9)	−0.00202 (9)	−0.00102 (9)
P1	0.0148 (3)	0.0191 (3)	0.0140 (3)	−0.0069 (2)	−0.0010 (2)	−0.0003 (2)
F1	0.0305 (9)	0.0228 (9)	0.0207 (8)	−0.0047 (7)	0.0020 (7)	−0.0019 (7)
F2	0.0597 (14)	0.0280 (10)	0.0167 (9)	−0.0118 (9)	0.0032 (9)	−0.0057 (7)
F3	0.0493 (12)	0.0416 (11)	0.0174 (9)	−0.0225 (10)	−0.0128 (8)	0.0045 (8)
F4	0.0237 (9)	0.0416 (11)	0.0290 (10)	−0.0067 (8)	−0.0095 (8)	0.0106 (8)
F5	0.0184 (8)	0.0305 (9)	0.0216 (8)	−0.0033 (7)	0.0003 (6)	0.0010 (7)
O1	0.0257 (11)	0.0286 (11)	0.0233 (10)	−0.0106 (9)	−0.0079 (8)	0.0014 (9)
O2	0.0416 (14)	0.0218 (11)	0.0388 (14)	−0.0118 (10)	0.0015 (11)	−0.0069 (10)
O3	0.0284 (11)	0.0310 (12)	0.0222 (11)	−0.0029 (9)	−0.0054 (9)	0.0007 (9)
O4	0.0379 (13)	0.0264 (11)	0.0218 (11)	−0.0098 (10)	−0.0083 (9)	0.0030 (9)
O5	0.0406 (14)	0.0228 (11)	0.0251 (11)	0.0018 (10)	0.0015 (10)	−0.0018 (9)
O6	0.0290 (11)	0.0296 (11)	0.0200 (10)	−0.0128 (9)	−0.0051 (8)	0.0012 (8)
O7	0.0228 (11)	0.0312 (12)	0.0276 (11)	−0.0112 (9)	−0.0065 (9)	0.0065 (9)
O8	0.0289 (13)	0.0386 (14)	0.0540 (17)	−0.0081 (11)	0.0100 (12)	−0.0237 (13)
O9	0.0336 (13)	0.0451 (14)	0.0220 (11)	−0.0191 (11)	−0.0063 (9)	0.0057 (10)
C1	0.0175 (12)	0.0177 (12)	0.0193 (12)	−0.0049 (10)	−0.0032 (10)	−0.0037 (10)
C2	0.0196 (13)	0.0227 (13)	0.0186 (12)	−0.0077 (10)	−0.0007 (10)	−0.0062 (10)
C3	0.0178 (13)	0.0263 (14)	0.0277 (15)	−0.0050 (11)	−0.0011 (11)	−0.0113 (12)
C4	0.0218 (14)	0.0222 (14)	0.0323 (16)	0.0001 (11)	−0.0083 (12)	−0.0083 (12)
C5	0.0284 (15)	0.0204 (14)	0.0304 (16)	−0.0036 (12)	−0.0077 (12)	0.0010 (12)
C6	0.0211 (13)	0.0208 (13)	0.0224 (13)	−0.0051 (11)	−0.0035 (10)	−0.0001 (11)
C7	0.0176 (12)	0.0178 (12)	0.0138 (11)	−0.0028 (10)	−0.0014 (9)	−0.0010 (9)
C8	0.0288 (15)	0.0218 (13)	0.0199 (13)	−0.0094 (11)	−0.0014 (11)	−0.0015 (11)
C9	0.0380 (18)	0.0224 (14)	0.0232 (15)	−0.0074 (13)	0.0009 (13)	0.0027 (11)
C10	0.0335 (17)	0.0284 (15)	0.0158 (13)	0.0016 (13)	−0.0024 (11)	0.0015 (11)
C11	0.0290 (15)	0.0291 (15)	0.0192 (13)	−0.0032 (12)	−0.0050 (11)	−0.0057 (11)
C12	0.0232 (13)	0.0204 (13)	0.0196 (13)	−0.0049 (11)	−0.0032 (10)	−0.0023 (10)
C13	0.0148 (11)	0.0172 (12)	0.0171 (12)	−0.0043 (9)	−0.0025 (9)	−0.0026 (9)
C14	0.0203 (13)	0.0178 (12)	0.0158 (12)	−0.0074 (10)	−0.0022 (10)	−0.0010 (9)
C15	0.0191 (13)	0.0246 (14)	0.0212 (13)	−0.0077 (11)	−0.0041 (10)	−0.0009 (11)
C16	0.0277 (15)	0.0254 (14)	0.0253 (15)	−0.0119 (12)	−0.0086 (12)	0.0075 (12)
C17	0.0324 (16)	0.0299 (16)	0.0278 (15)	−0.0185 (13)	−0.0071 (13)	0.0089 (12)
C18	0.0234 (14)	0.0317 (16)	0.0310 (16)	−0.0161 (13)	−0.0037 (12)	0.0039 (13)
C19	0.0196 (13)	0.0216 (13)	0.0277 (14)	−0.0096 (11)	−0.0042 (11)	0.0001 (11)
C20	0.0185 (12)	0.0190 (12)	0.0183 (12)	−0.0067 (10)	−0.0025 (10)	−0.0032 (10)
C21	0.0228 (15)	0.0383 (18)	0.0352 (18)	0.0006 (13)	−0.0047 (13)	−0.0181 (15)
C22	0.0331 (18)	0.050 (2)	0.040 (2)	−0.0014 (16)	−0.0035 (15)	−0.0302 (18)
C23	0.0327 (17)	0.0384 (18)	0.0231 (15)	−0.0135 (14)	−0.0041 (12)	−0.0118 (13)
C24	0.0234 (14)	0.0300 (15)	0.0269 (15)	−0.0082 (12)	−0.0078 (12)	−0.0053 (12)
C25	0.0196 (13)	0.0240 (14)	0.0235 (14)	−0.0046 (11)	−0.0038 (10)	−0.0066 (11)
C26	0.0182 (12)	0.0239 (13)	0.0146 (11)	−0.0116 (10)	−0.0012 (9)	−0.0003 (10)
C27	0.0245 (14)	0.0209 (13)	0.0181 (12)	−0.0112 (11)	−0.0005 (10)	−0.0006 (10)
C28	0.0360 (16)	0.0250 (14)	0.0163 (13)	−0.0166 (13)	0.0000 (11)	−0.0021 (11)
C29	0.0337 (16)	0.0307 (15)	0.0195 (13)	−0.0208 (13)	−0.0078 (12)	0.0029 (11)
C30	0.0183 (13)	0.0292 (15)	0.0244 (14)	−0.0122 (11)	−0.0065 (11)	0.0071 (11)
C31	0.0172 (12)	0.0258 (14)	0.0179 (12)	−0.0105 (11)	0.0000 (10)	0.0010 (10)
C32	0.0205 (13)	0.0201 (12)	0.0158 (12)	−0.0078 (10)	0.0005 (10)	−0.0003 (10)
C33	0.0249 (14)	0.0248 (14)	0.0222 (14)	−0.0082 (11)	−0.0046 (11)	0.0016 (11)
C34	0.0318 (16)	0.0291 (16)	0.0267 (16)	−0.0065 (13)	−0.0073 (13)	0.0093 (13)
C35	0.0404 (19)	0.0198 (14)	0.0276 (16)	−0.0057 (13)	−0.0023 (13)	0.0043 (12)
C36	0.0374 (17)	0.0229 (14)	0.0217 (14)	−0.0123 (13)	0.0018 (12)	−0.0033 (11)
C37	0.0258 (14)	0.0239 (14)	0.0193 (13)	−0.0093 (11)	−0.0010 (11)	−0.0044 (11)
C38	0.0167 (12)	0.0208 (12)	0.0160 (12)	−0.0070 (10)	0.0002 (9)	0.0008 (10)
C39	0.0183 (12)	0.0211 (13)	0.0195 (13)	−0.0070 (10)	−0.0025 (10)	0.0012 (10)
C40	0.0154 (12)	0.0251 (14)	0.0235 (14)	−0.0087 (10)	−0.0016 (10)	0.0032 (11)
C41	0.0199 (13)	0.0250 (14)	0.0249 (14)	−0.0071 (11)	0.0038 (11)	0.0013 (11)
C42	0.0262 (14)	0.0265 (14)	0.0167 (13)	−0.0086 (12)	0.0025 (11)	−0.0021 (11)
C43	0.0205 (13)	0.0239 (13)	0.0191 (13)	−0.0095 (11)	−0.0015 (10)	−0.0012 (10)
C44	0.0191 (12)	0.0180 (12)	0.0223 (13)	−0.0084 (10)	−0.0012 (10)	−0.0005 (10)
C45	0.0225 (13)	0.0242 (14)	0.0217 (13)	−0.0096 (11)	−0.0007 (11)	0.0002 (11)
C46	0.0180 (12)	0.0195 (12)	0.0214 (13)	−0.0045 (10)	0.0010 (10)	−0.0014 (10)
C47	0.0208 (13)	0.0176 (12)	0.0227 (13)	−0.0056 (10)	−0.0018 (10)	0.0002 (10)
C48	0.0219 (13)	0.0213 (13)	0.0159 (12)	−0.0060 (11)	−0.0003 (10)	0.0010 (10)
C49	0.0168 (12)	0.0210 (13)	0.0212 (13)	−0.0086 (10)	−0.0016 (10)	−0.0020 (10)
C50	0.0173 (12)	0.0238 (14)	0.0252 (14)	−0.0100 (11)	−0.0010 (10)	0.0000 (11)
C51	0.0224 (14)	0.0276 (15)	0.0280 (15)	−0.0112 (12)	0.0013 (11)	−0.0048 (12)
C52	0.0202 (13)	0.0279 (14)	0.0225 (14)	−0.0120 (11)	−0.0011 (10)	−0.0027 (11)
Cl1A	0.209 (5)	0.0446 (14)	0.092 (2)	0.009 (2)	−0.112 (3)	−0.0128 (14)
Cl2A	0.068 (2)	0.097 (3)	0.120 (3)	−0.049 (2)	0.006 (2)	−0.047 (2)
Cl3A	0.060 (2)	0.275 (7)	0.0578 (19)	−0.041 (3)	0.0028 (15)	−0.055 (3)
C53A	0.116 (11)	0.045 (5)	0.055 (6)	−0.042 (7)	0.034 (7)	−0.015 (5)

### Geometric parameters (Å, °)

**Table:**

Ru1—C45	1.897 (3)	C13—H13A	0.9700
Ru1—C44	1.922 (3)	C13—H13B	0.9700
Ru1—C46	1.931 (3)	C14—C15	1.388 (4)
Ru1—As1	2.4210 (3)	C14—C19	1.395 (4)
Ru1—Ru2	2.8653 (3)	C15—C16	1.392 (4)
Ru1—Ru3	2.8730 (3)	C15—H15A	0.9300
Ru2—C48	1.894 (3)	C16—C17	1.384 (5)
Ru2—C47	1.927 (3)	C16—H16A	0.9300
Ru2—C49	1.940 (3)	C17—C18	1.391 (5)
Ru2—As2	2.4243 (3)	C17—H17A	0.9300
Ru2—Ru3	2.8418 (3)	C18—C19	1.389 (4)
Ru3—C51	1.887 (3)	C18—H18A	0.9300
Ru3—C50	1.931 (3)	C19—H19A	0.9300
Ru3—C52	1.946 (3)	C20—C21	1.389 (4)
Ru3—P1	2.3367 (7)	C20—C25	1.392 (4)
As1—C7	1.938 (3)	C21—C22	1.390 (5)
As1—C1	1.951 (3)	C21—H21A	0.9300
As1—C13	1.964 (3)	C22—C23	1.385 (5)
As2—C14	1.937 (3)	C22—H22A	0.9300
As2—C20	1.939 (3)	C23—C24	1.369 (5)
As2—C13	1.958 (3)	C23—H23A	0.9300
P1—C32	1.835 (3)	C24—C25	1.396 (4)
P1—C38	1.839 (3)	C24—H24A	0.9300
P1—C26	1.844 (3)	C25—H25A	0.9300
F1—C27	1.339 (3)	C26—C31	1.393 (4)
F2—C28	1.337 (4)	C26—C27	1.395 (4)
F3—C29	1.333 (3)	C27—C28	1.390 (4)
F4—C30	1.337 (4)	C28—C29	1.377 (5)
F5—C31	1.346 (3)	C29—C30	1.384 (5)
O1—C44	1.148 (4)	C30—C31	1.382 (4)
O2—C45	1.142 (4)	C32—C33	1.390 (4)
O3—C46	1.150 (4)	C32—C37	1.405 (4)
O4—C47	1.139 (4)	C33—C34	1.390 (4)
O5—C48	1.146 (4)	C33—H33A	0.9300
O6—C49	1.143 (4)	C34—C35	1.378 (5)
O7—C50	1.142 (4)	C34—H34A	0.9300
O8—C51	1.140 (4)	C35—C36	1.399 (5)
O9—C52	1.136 (4)	C35—H35A	0.9300
C1—C2	1.392 (4)	C36—C37	1.385 (4)
C1—C6	1.398 (4)	C36—H36A	0.9300
C2—C3	1.399 (4)	C37—H37A	0.9300
C2—H2A	0.9300	C38—C43	1.394 (4)
C3—C4	1.386 (5)	C38—C39	1.396 (4)
C3—H3A	0.9300	C39—C40	1.391 (4)
C4—C5	1.392 (5)	C39—H39A	0.9300
C4—H4A	0.9300	C40—C41	1.386 (4)
C5—C6	1.391 (4)	C40—H40A	0.9300
C5—H5A	0.9300	C41—C42	1.387 (4)
C6—H6A	0.9300	C41—H41A	0.9300
C7—C12	1.392 (4)	C42—C43	1.396 (4)
C7—C8	1.398 (4)	C42—H42A	0.9300
C8—C9	1.395 (4)	C43—H43A	0.9300
C8—H8A	0.9300	Cl1A—C53A	1.725 (11)
C9—C10	1.388 (5)	Cl2A—C53A	1.723 (14)
C9—H9A	0.9300	Cl3A—C53A	1.731 (10)
C10—C11	1.388 (5)	C53A—H53A	0.9800
C10—H10A	0.9300	Cl1B—C53B	1.794 (14)
C11—C12	1.394 (4)	Cl2B—C53B	1.731 (16)
C11—H11A	0.9300	Cl3B—C53B	1.644 (15)
C12—H12A	0.9300	C53B—H53B	0.9800
			
C45—Ru1—C44	87.69 (13)	C14—C15—C16	120.2 (3)
C45—Ru1—C46	92.37 (13)	C14—C15—H15A	119.9
C44—Ru1—C46	172.77 (12)	C16—C15—H15A	119.9
C45—Ru1—As1	99.18 (9)	C17—C16—C15	119.8 (3)
C44—Ru1—As1	94.51 (8)	C17—C16—H16A	120.1
C46—Ru1—As1	92.62 (8)	C15—C16—H16A	120.1
C45—Ru1—Ru2	170.93 (9)	C16—C17—C18	120.3 (3)
C44—Ru1—Ru2	93.53 (9)	C16—C17—H17A	119.9
C46—Ru1—Ru2	85.29 (9)	C18—C17—H17A	119.9
As1—Ru1—Ru2	89.691 (10)	C19—C18—C17	120.0 (3)
C45—Ru1—Ru3	111.96 (9)	C19—C18—H18A	120.0
C44—Ru1—Ru3	83.03 (9)	C17—C18—H18A	120.0
C46—Ru1—Ru3	90.25 (8)	C18—C19—C14	119.8 (3)
As1—Ru1—Ru3	148.582 (12)	C18—C19—H19A	120.1
Ru2—Ru1—Ru3	59.370 (7)	C14—C19—H19A	120.1
C48—Ru2—C47	93.06 (12)	C21—C20—C25	119.2 (3)
C48—Ru2—C49	92.85 (12)	C21—C20—As2	117.6 (2)
C47—Ru2—C49	174.09 (12)	C25—C20—As2	123.1 (2)
C48—Ru2—As2	102.74 (9)	C20—C21—C22	120.2 (3)
C47—Ru2—As2	88.81 (9)	C20—C21—H21A	119.9
C49—Ru2—As2	89.79 (8)	C22—C21—H21A	119.9
C48—Ru2—Ru3	101.06 (9)	C23—C22—C21	120.1 (3)
C47—Ru2—Ru3	80.96 (9)	C23—C22—H22A	119.9
C49—Ru2—Ru3	98.00 (8)	C21—C22—H22A	119.9
As2—Ru2—Ru3	154.528 (12)	C24—C23—C22	120.0 (3)
C48—Ru2—Ru1	159.52 (9)	C24—C23—H23A	120.0
C47—Ru2—Ru1	92.48 (9)	C22—C23—H23A	120.0
C49—Ru2—Ru1	82.00 (9)	C23—C24—C25	120.4 (3)
As2—Ru2—Ru1	97.077 (10)	C23—C24—H24A	119.8
Ru3—Ru2—Ru1	60.450 (7)	C25—C24—H24A	119.8
C51—Ru3—C50	87.52 (14)	C20—C25—C24	120.0 (3)
C51—Ru3—C52	91.29 (14)	C20—C25—H25A	120.0
C50—Ru3—C52	173.07 (12)	C24—C25—H25A	120.0
C51—Ru3—P1	100.25 (9)	C31—C26—C27	115.3 (3)
C50—Ru3—P1	93.42 (9)	C31—C26—P1	120.1 (2)
C52—Ru3—P1	93.52 (9)	C27—C26—P1	123.6 (2)
C51—Ru3—Ru2	97.64 (9)	F1—C27—C28	116.5 (3)
C50—Ru3—Ru2	76.95 (8)	F1—C27—C26	121.0 (3)
C52—Ru3—Ru2	96.46 (8)	C28—C27—C26	122.5 (3)
P1—Ru3—Ru2	159.28 (2)	F2—C28—C29	119.7 (3)
C51—Ru3—Ru1	157.38 (9)	F2—C28—C27	120.3 (3)
C50—Ru3—Ru1	91.23 (9)	C29—C28—C27	120.0 (3)
C52—Ru3—Ru1	87.24 (9)	F3—C29—C28	120.7 (3)
P1—Ru3—Ru1	102.374 (19)	F3—C29—C30	119.8 (3)
Ru2—Ru3—Ru1	60.180 (7)	C28—C29—C30	119.4 (3)
C7—As1—C1	101.81 (11)	F4—C30—C31	120.7 (3)
C7—As1—C13	104.16 (11)	F4—C30—C29	120.0 (3)
C1—As1—C13	98.63 (11)	C31—C30—C29	119.3 (3)
C7—As1—Ru1	117.89 (8)	F5—C31—C30	116.5 (3)
C1—As1—Ru1	117.30 (8)	F5—C31—C26	119.9 (2)
C13—As1—Ru1	114.39 (8)	C30—C31—C26	123.4 (3)
C14—As2—C20	99.53 (12)	C33—C32—C37	118.8 (3)
C14—As2—C13	103.74 (12)	C33—C32—P1	119.9 (2)
C20—As2—C13	102.41 (11)	C37—C32—P1	120.9 (2)
C14—As2—Ru2	118.35 (8)	C34—C33—C32	120.6 (3)
C20—As2—Ru2	117.46 (8)	C34—C33—H33A	119.7
C13—As2—Ru2	113.05 (8)	C32—C33—H33A	119.7
C32—P1—C38	97.60 (13)	C35—C34—C33	120.4 (3)
C32—P1—C26	104.34 (13)	C35—C34—H34A	119.8
C38—P1—C26	103.31 (13)	C33—C34—H34A	119.8
C32—P1—Ru3	122.83 (10)	C34—C35—C36	119.8 (3)
C38—P1—Ru3	117.20 (10)	C34—C35—H35A	120.1
C26—P1—Ru3	109.21 (9)	C36—C35—H35A	120.1
C2—C1—C6	119.5 (3)	C37—C36—C35	120.0 (3)
C2—C1—As1	121.4 (2)	C37—C36—H36A	120.0
C6—C1—As1	119.0 (2)	C35—C36—H36A	120.0
C1—C2—C3	120.0 (3)	C36—C37—C32	120.4 (3)
C1—C2—H2A	120.0	C36—C37—H37A	119.8
C3—C2—H2A	120.0	C32—C37—H37A	119.8
C4—C3—C2	120.4 (3)	C43—C38—C39	118.8 (3)
C4—C3—H3A	119.8	C43—C38—P1	117.6 (2)
C2—C3—H3A	119.8	C39—C38—P1	123.4 (2)
C3—C4—C5	119.7 (3)	C40—C39—C38	120.4 (3)
C3—C4—H4A	120.2	C40—C39—H39A	119.8
C5—C4—H4A	120.2	C38—C39—H39A	119.8
C6—C5—C4	120.3 (3)	C41—C40—C39	120.5 (3)
C6—C5—H5A	119.8	C41—C40—H40A	119.8
C4—C5—H5A	119.8	C39—C40—H40A	119.8
C5—C6—C1	120.2 (3)	C40—C41—C42	119.8 (3)
C5—C6—H6A	119.9	C40—C41—H41A	120.1
C1—C6—H6A	119.9	C42—C41—H41A	120.1
C12—C7—C8	120.0 (3)	C41—C42—C43	119.8 (3)
C12—C7—As1	117.6 (2)	C41—C42—H42A	120.1
C8—C7—As1	122.4 (2)	C43—C42—H42A	120.1
C9—C8—C7	119.5 (3)	C38—C43—C42	120.8 (3)
C9—C8—H8A	120.3	C38—C43—H43A	119.6
C7—C8—H8A	120.3	C42—C43—H43A	119.6
C10—C9—C8	120.5 (3)	O1—C44—Ru1	173.4 (3)
C10—C9—H9A	119.8	O2—C45—Ru1	176.8 (3)
C8—C9—H9A	119.8	O3—C46—Ru1	174.1 (3)
C11—C10—C9	119.9 (3)	O4—C47—Ru2	172.9 (3)
C11—C10—H10A	120.1	O5—C48—Ru2	178.1 (3)
C9—C10—H10A	120.1	O6—C49—Ru2	173.8 (3)
C10—C11—C12	120.2 (3)	O7—C50—Ru3	171.5 (3)
C10—C11—H11A	119.9	O8—C51—Ru3	177.7 (3)
C12—C11—H11A	119.9	O9—C52—Ru3	173.4 (3)
C7—C12—C11	120.0 (3)	Cl2A—C53A—Cl1A	111.5 (6)
C7—C12—H12A	120.0	Cl2A—C53A—Cl3A	112.4 (7)
C11—C12—H12A	120.0	Cl1A—C53A—Cl3A	109.9 (7)
As2—C13—As1	111.64 (13)	Cl2A—C53A—H53A	107.6
As2—C13—H13A	109.3	Cl1A—C53A—H53A	107.6
As1—C13—H13A	109.3	Cl3A—C53A—H53A	107.6
As2—C13—H13B	109.3	Cl3B—C53B—Cl2B	116.4 (10)
As1—C13—H13B	109.3	Cl3B—C53B—Cl1B	114.4 (9)
H13A—C13—H13B	108.0	Cl2B—C53B—Cl1B	107.6 (9)
C15—C14—C19	119.9 (3)	Cl3B—C53B—H53B	105.9
C15—C14—As2	119.8 (2)	Cl2B—C53B—H53B	105.9
C19—C14—As2	120.3 (2)	Cl1B—C53B—H53B	105.9
			
C44—Ru1—Ru2—C48	52.3 (3)	C13—As1—C1—C6	100.1 (2)
C46—Ru1—Ru2—C48	−120.5 (3)	Ru1—As1—C1—C6	−23.2 (3)
As1—Ru1—Ru2—C48	146.8 (2)	C6—C1—C2—C3	0.0 (4)
Ru3—Ru1—Ru2—C48	−27.4 (2)	As1—C1—C2—C3	177.9 (2)
C44—Ru1—Ru2—C47	157.91 (12)	C1—C2—C3—C4	−0.2 (4)
C46—Ru1—Ru2—C47	−14.94 (12)	C2—C3—C4—C5	−0.1 (5)
As1—Ru1—Ru2—C47	−107.60 (9)	C3—C4—C5—C6	0.6 (5)
Ru3—Ru1—Ru2—C47	78.15 (9)	C4—C5—C6—C1	−0.8 (5)
C44—Ru1—Ru2—C49	−24.20 (12)	C2—C1—C6—C5	0.5 (4)
C46—Ru1—Ru2—C49	162.95 (12)	As1—C1—C6—C5	−177.5 (2)
As1—Ru1—Ru2—C49	70.29 (8)	C1—As1—C7—C12	72.1 (2)
Ru3—Ru1—Ru2—C49	−103.96 (8)	C13—As1—C7—C12	174.2 (2)
C44—Ru1—Ru2—As2	−112.99 (8)	Ru1—As1—C7—C12	−57.8 (2)
C46—Ru1—Ru2—As2	74.16 (8)	C1—As1—C7—C8	−110.1 (2)
As1—Ru1—Ru2—As2	−18.492 (12)	C13—As1—C7—C8	−7.9 (3)
Ru3—Ru1—Ru2—As2	167.256 (12)	Ru1—As1—C7—C8	120.0 (2)
C44—Ru1—Ru2—Ru3	79.75 (8)	C12—C7—C8—C9	−0.5 (5)
C46—Ru1—Ru2—Ru3	−93.09 (8)	As1—C7—C8—C9	−178.2 (2)
As1—Ru1—Ru2—Ru3	174.251 (11)	C7—C8—C9—C10	−0.5 (5)
C48—Ru2—Ru3—C51	−14.21 (13)	C8—C9—C10—C11	0.6 (5)
C47—Ru2—Ru3—C51	77.17 (14)	C9—C10—C11—C12	0.2 (5)
C49—Ru2—Ru3—C51	−108.71 (13)	C8—C7—C12—C11	1.3 (4)
As2—Ru2—Ru3—C51	144.65 (11)	As1—C7—C12—C11	179.2 (2)
Ru1—Ru2—Ru3—C51	175.25 (10)	C10—C11—C12—C7	−1.2 (5)
C48—Ru2—Ru3—C50	71.44 (13)	C14—As2—C13—As1	−106.55 (14)
C47—Ru2—Ru3—C50	162.82 (13)	C20—As2—C13—As1	150.25 (13)
C49—Ru2—Ru3—C50	−23.07 (13)	Ru2—As2—C13—As1	22.89 (15)
As2—Ru2—Ru3—C50	−129.70 (10)	C7—As1—C13—As2	88.09 (14)
Ru1—Ru2—Ru3—C50	−99.10 (9)	C1—As1—C13—As2	−167.33 (13)
C48—Ru2—Ru3—C52	−106.39 (13)	Ru1—As1—C13—As2	−42.01 (15)
C47—Ru2—Ru3—C52	−15.01 (13)	C20—As2—C14—C15	−115.7 (2)
C49—Ru2—Ru3—C52	159.11 (13)	C13—As2—C14—C15	138.9 (2)
As2—Ru2—Ru3—C52	52.47 (10)	Ru2—As2—C14—C15	12.8 (3)
Ru1—Ru2—Ru3—C52	83.07 (9)	C20—As2—C14—C19	63.6 (3)
C48—Ru2—Ru3—P1	135.34 (10)	C13—As2—C14—C19	−41.8 (3)
C47—Ru2—Ru3—P1	−133.28 (10)	Ru2—As2—C14—C19	−167.9 (2)
C49—Ru2—Ru3—P1	40.83 (10)	C19—C14—C15—C16	2.0 (4)
As2—Ru2—Ru3—P1	−65.80 (7)	As2—C14—C15—C16	−178.6 (2)
Ru1—Ru2—Ru3—P1	−35.20 (6)	C14—C15—C16—C17	−0.1 (5)
C48—Ru2—Ru3—Ru1	170.54 (9)	C15—C16—C17—C18	−1.0 (5)
C47—Ru2—Ru3—Ru1	−98.08 (9)	C16—C17—C18—C19	0.1 (5)
C49—Ru2—Ru3—Ru1	76.04 (9)	C17—C18—C19—C14	1.9 (5)
As2—Ru2—Ru3—Ru1	−30.60 (3)	C15—C14—C19—C18	−2.9 (5)
C45—Ru1—Ru3—C51	164.7 (3)	As2—C14—C19—C18	177.7 (2)
C44—Ru1—Ru3—C51	−110.6 (3)	C14—As2—C20—C21	84.5 (3)
C46—Ru1—Ru3—C51	72.0 (3)	C13—As2—C20—C21	−169.1 (3)
As1—Ru1—Ru3—C51	−23.4 (3)	Ru2—As2—C20—C21	−44.6 (3)
Ru2—Ru1—Ru3—C51	−12.3 (3)	C14—As2—C20—C25	−93.0 (3)
C45—Ru1—Ru3—C50	−108.79 (13)	C13—As2—C20—C25	13.4 (3)
C44—Ru1—Ru3—C50	−24.13 (12)	Ru2—As2—C20—C25	137.9 (2)
C46—Ru1—Ru3—C50	158.55 (12)	C25—C20—C21—C22	0.5 (5)
As1—Ru1—Ru3—C50	63.10 (9)	As2—C20—C21—C22	−177.1 (3)
Ru2—Ru1—Ru3—C50	74.18 (9)	C20—C21—C22—C23	0.4 (6)
C45—Ru1—Ru3—C52	77.98 (13)	C21—C22—C23—C24	−0.7 (6)
C44—Ru1—Ru3—C52	162.64 (12)	C22—C23—C24—C25	0.1 (5)
C46—Ru1—Ru3—C52	−14.68 (12)	C21—C20—C25—C24	−1.1 (5)
As1—Ru1—Ru3—C52	−110.13 (9)	As2—C20—C25—C24	176.4 (2)
Ru2—Ru1—Ru3—C52	−99.05 (9)	C23—C24—C25—C20	0.8 (5)
C45—Ru1—Ru3—P1	−15.02 (10)	C32—P1—C26—C31	153.1 (2)
C44—Ru1—Ru3—P1	69.64 (8)	C38—P1—C26—C31	51.5 (2)
C46—Ru1—Ru3—P1	−107.68 (9)	Ru3—P1—C26—C31	−73.9 (2)
As1—Ru1—Ru3—P1	156.87 (3)	C32—P1—C26—C27	−38.5 (3)
Ru2—Ru1—Ru3—P1	167.95 (2)	C38—P1—C26—C27	−140.1 (2)
C45—Ru1—Ru3—Ru2	177.03 (10)	Ru3—P1—C26—C27	94.4 (2)
C44—Ru1—Ru3—Ru2	−98.31 (8)	C31—C26—C27—F1	175.4 (2)
C46—Ru1—Ru3—Ru2	84.37 (9)	P1—C26—C27—F1	6.6 (4)
As1—Ru1—Ru3—Ru2	−11.08 (2)	C31—C26—C27—C28	−1.4 (4)
C45—Ru1—As1—C7	94.63 (13)	P1—C26—C27—C28	−170.2 (2)
C44—Ru1—As1—C7	6.24 (13)	F1—C27—C28—F2	1.4 (4)
C46—Ru1—As1—C7	−172.54 (13)	C26—C27—C28—F2	178.4 (3)
Ru2—Ru1—As1—C7	−87.27 (9)	F1—C27—C28—C29	−177.4 (3)
Ru3—Ru1—As1—C7	−77.76 (10)	C26—C27—C28—C29	−0.4 (4)
C45—Ru1—As1—C1	−27.62 (13)	F2—C28—C29—F3	1.0 (4)
C44—Ru1—As1—C1	−116.01 (13)	C27—C28—C29—F3	179.9 (3)
C46—Ru1—As1—C1	65.21 (13)	F2—C28—C29—C30	−176.6 (3)
Ru2—Ru1—As1—C1	150.47 (9)	C27—C28—C29—C30	2.3 (4)
Ru3—Ru1—As1—C1	159.99 (9)	F3—C29—C30—F4	−0.5 (4)
C45—Ru1—As1—C13	−142.42 (13)	C28—C29—C30—F4	177.1 (3)
C44—Ru1—As1—C13	129.19 (12)	F3—C29—C30—C31	−179.8 (3)
C46—Ru1—As1—C13	−49.59 (12)	C28—C29—C30—C31	−2.1 (4)
Ru2—Ru1—As1—C13	35.68 (9)	F4—C30—C31—F5	−2.5 (4)
Ru3—Ru1—As1—C13	45.19 (9)	C29—C30—C31—F5	176.8 (3)
C48—Ru2—As2—C14	−52.19 (13)	F4—C30—C31—C26	−179.0 (3)
C47—Ru2—As2—C14	−145.06 (13)	C29—C30—C31—C26	0.2 (4)
C49—Ru2—As2—C14	40.68 (13)	C27—C26—C31—F5	−174.9 (2)
Ru3—Ru2—As2—C14	149.09 (10)	P1—C26—C31—F5	−5.7 (4)
Ru1—Ru2—As2—C14	122.59 (9)	C27—C26—C31—C30	1.5 (4)
C48—Ru2—As2—C20	67.30 (13)	P1—C26—C31—C30	170.8 (2)
C47—Ru2—As2—C20	−25.57 (13)	C38—P1—C32—C33	−114.9 (2)
C49—Ru2—As2—C20	160.18 (13)	C26—P1—C32—C33	139.2 (2)
Ru3—Ru2—As2—C20	−91.42 (10)	Ru3—P1—C32—C33	14.5 (3)
Ru1—Ru2—As2—C20	−117.92 (9)	C38—P1—C32—C37	58.8 (3)
C48—Ru2—As2—C13	−173.72 (12)	C26—P1—C32—C37	−47.1 (3)
C47—Ru2—As2—C13	93.41 (12)	Ru3—P1—C32—C37	−171.80 (19)
C49—Ru2—As2—C13	−80.85 (12)	C37—C32—C33—C34	0.0 (4)
Ru3—Ru2—As2—C13	27.56 (9)	P1—C32—C33—C34	173.9 (2)
Ru1—Ru2—As2—C13	1.06 (9)	C32—C33—C34—C35	−0.8 (5)
C51—Ru3—P1—C32	−136.81 (15)	C33—C34—C35—C36	0.9 (5)
C50—Ru3—P1—C32	135.09 (14)	C34—C35—C36—C37	−0.2 (5)
C52—Ru3—P1—C32	−44.86 (14)	C35—C36—C37—C32	−0.5 (5)
Ru2—Ru3—P1—C32	73.88 (12)	C33—C32—C37—C36	0.6 (4)
Ru1—Ru3—P1—C32	43.08 (11)	P1—C32—C37—C36	−173.2 (2)
C51—Ru3—P1—C38	−16.24 (14)	C32—P1—C38—C43	96.1 (2)
C50—Ru3—P1—C38	−104.34 (14)	C26—P1—C38—C43	−157.1 (2)
C52—Ru3—P1—C38	75.70 (14)	Ru3—P1—C38—C43	−37.0 (3)
Ru2—Ru3—P1—C38	−165.55 (10)	C32—P1—C38—C39	−77.2 (3)
Ru1—Ru3—P1—C38	163.65 (10)	C26—P1—C38—C39	29.6 (3)
C51—Ru3—P1—C26	100.71 (14)	Ru3—P1—C38—C39	149.7 (2)
C50—Ru3—P1—C26	12.61 (13)	C43—C38—C39—C40	−0.2 (4)
C52—Ru3—P1—C26	−167.34 (13)	P1—C38—C39—C40	173.0 (2)
Ru2—Ru3—P1—C26	−48.60 (12)	C38—C39—C40—C41	0.5 (4)
Ru1—Ru3—P1—C26	−79.40 (10)	C39—C40—C41—C42	−0.9 (5)
C7—As1—C1—C2	28.7 (3)	C40—C41—C42—C43	1.0 (5)
C13—As1—C1—C2	−77.9 (2)	C39—C38—C43—C42	0.3 (4)
Ru1—As1—C1—C2	158.9 (2)	P1—C38—C43—C42	−173.3 (2)
C7—As1—C1—C6	−153.4 (2)	C41—C42—C43—C38	−0.7 (5)

### Hydrogen-bond geometry (Å, °)

**Table:**

Cg1 and Cg2 are the centroids of the C1–C6 and C32–C37 benzene rings, respectively.

**Table:**

*D*—H···*A*	*D*—H	H···*A*	*D*···*A*	*D*—H···*A*
C10—H10A···O5^i^	0.93	2.58	3.469 (4)	160
C12—H12A···F2^ii^	0.93	2.48	3.274 (3)	143
C22—H22A···O4^iii^	0.93	2.59	3.451 (5)	154
C34—H34A···O4^iv^	0.93	2.49	3.201 (4)	134
C39—H39A···Cg1^v^	0.93	2.93	3.749 (3)	148
C41—H41A···Cg2^vi^	0.93	2.73	3.607 (3)	157

Symmetry codes: (i) −*x*+1, −*y*+1, −*z*; (ii) −*x*+1, −*y*, −*z*; (iii) −*x*+1, −*y*+1, −*z*+1; (iv) −*x*+1, −*y*, −*z*+1; (v) *x*−1, *y*, *z*; (vi) −*x*, −*y*, −*z*+1.
